# Community-based intervention for prevention and management of chronic obstructive pulmonary disease in Nepal (COBIN-P trial): study protocol for a cluster-randomized controlled trial

**DOI:** 10.1186/s13063-021-05447-7

**Published:** 2021-07-21

**Authors:** Tara Ballav Adhikari, Dinesh Neupane, Arjun Karki, Arne Drews, Brendan Cooper, Marieann Högman, Torben Sigsgaard, Per Kallestrup

**Affiliations:** 1 Department of Public Health, Section for Global Health, Aarhus University, Aarhus, Denmark; 2 COBIN Project, Nepal Development Society, Bharatpur, Chitwan Nepal; 3 Welch Center for Prevention, Epidemiology, and Clinical Research, Department of Epidemiology, Johns Hopkins University, Baltimore, MD USA; 4 Department of Pulmonary, Critical Care and Sleep Medicine, HAMS Hospital, Kathmandu, Nepal; 5 Nepalmed, Leipzig, Germany; 6 Lung Function and Sleep, University Hospitals Birmingham, Birmingham, UK; 7 Department of Medical Sciences, Respiratory, Allergy and Sleep Research, Uppsala University, Uppsala, Sweden; 8 Department of Public Health, Section for Environment, Occupation & Health, Aarhus University, Aarhus, Denmark

**Keywords:** COPD, Community health workers, Nepal, Female Community Health Volunteers, Cluster randomized controlled trial

## Abstract

**Background:**

Chronic obstructive pulmonary disease (COPD) is one of the leading causes of death worldwide and the commonest of non-communicable diseases (NCDs) in Nepal. Risk factors like indoor and outdoor air pollution, a high prevalence of smoking, and the lack of awareness of COPD make it a serious public health concern. However, no attempt has been made in Nepal to estimate its burden and address the disease at the community level.

**Method:**

This study aims to evaluate the effect of a community-based health educational intervention administered by Female Community Health Volunteers (FCHVs) on the prevention and management of COPD. An open-label, two-group, community-based, cluster-randomized controlled trial will be implemented in the semi-urban area of Pokhara Metropolitan city (former Lekhnath Municipality) located in the Kaski district of Nepal. The estimated sample size of the intervention will be 1143. The unit of randomization is the ward (administrative unit) of the study area. The follow-up survey will be conducted immediately after 12 months of FCHVs-led interventions. The difference in the rate of decline of forced expiratory volume in 1 s (FEV_1_) and FEV_1_/FVC (forced vital capacity) ratio are the primary outcomes and the change in the proportion of modifiable risk factors of COPD, health-related quality of life scores, and change in knowledge of COPD will be secondary outcomes.

**Discussion:**

This study will estimate the burden of COPD, the magnitude of risk factors and generate evidence to mobilize community health workers for COPD prevention and management at the community level in Nepal.

**Trial registration:**

ClinicalTrials.gov NCT03797768. Registered on January 9, 2019.

## Background

Globally, non-communicable diseases (NCDs) are the leading causes of morbidity and mortality, accounting for the deaths of 41 million people annually [1]. Nearly three-quarters of NCDs-related global deaths occur in low- and middle-income countries (LMICs), like Nepal [2].

As of 2017, the Global Burden of Disease (GBD) study estimated that about 3.9 million people worldwide died of COPD, which is 5.7% of all deaths [1]. Far earlier than the World Health Organization (WHO) projection COPD to be the third most important cause of death worldwide by 2030 [3], the current estimation of the GBD study 2017 ranks it as the second killer disease globally [1].

The Global Initiative for Chronic Obstructive Lung Disease (GOLD) defines COPD as “a common, preventable and treatable disease that is characterized by persistent respiratory symptoms and airflow limitation due to airway and/or alveolar abnormalities usually caused by significant exposure to noxious particles or gases” [4]. COPD results from the interaction between genetic and environmental factors, including exposure to tobacco smoke, indoor and outdoor air pollution, and exposure to organic and inorganic dust, fumes, and gases [5].

WHO reports that 90% of COPD accounted for deaths occurs in LMICs [6]. Furthermore, COPD-associated mortality is predicted to increase by 160% in the Southeast Asian region in the coming decades [7]. COPD is one of the commonest forms of NCDs among the Nepalese adult population contributing to more than 40% of NCD cases among out-patients [8]. Such a high burden of COPD in the country has been attributed to the widespread use of traditional cooking stoves, mainly in the rural areas, and the burning of solid biomass fuels like animal dung, crop residue, and wood [9]. The national census of 2011 estimated that 75% of Nepalese populations use biomass fuels in poorly ventilated housing conditions [10]. Likewise, 27% of Nepali men (15–64 years) smoke tobacco while women (15–64 years) are also known to smoke (10%) considerably higher by Southeast Asian average [11]. The WHO STEPS survey in 2019 estimated that more than 33% of the Nepalese adult population (15–64 years) is exposed to second-hand smoking at home [12]. Similarly, a recent study in eastern Nepal revealed that the level of inadequate health literacy was 77% among patients with COPD [13].

These statistics suggest that addressing smoking along with indoor and outdoor air pollution remains the cornerstone of COPD prevention in Nepal. However, the COPD diagnosis with spirometry, its treatment, and management is usually limited to specialized tertiary level health facilities. It is beyond the reach of district health facilities in resource-limited countries [14]. On the other hand, more simple measures like spreading awareness on recognizing cough, sputum production, and breathlessness as essential symptoms have the potential to go a long way as a necessary aspect of public health care of COPD [15].

Most community-based programs studied previously focused particularly on pulmonary rehabilitation, smoking cessation counseling, improving health-related quality of life, self-management counseling, reducing hospital admissions, delivered mainly by physicians, nurses, and physiotherapists [16–22]. The FRESH Air project study in Uganda involved midwives, but the intervention included the delivering of health education messages on the dangers of biomass smoke to pregnant women and post-natal mothers through the regular ante- and post-natal clinics [23]. A published protocol intended to involve community health workers (CHWs) for supporting in the self-management of COPD cases in Bhaktapur district of central Nepal [24]. Likewise, a study reported that CHWs were invited for inputs during consultative meetings while designing probable self-management interventions in multimorbid COPD cases in rural Nepal [25]. However, none of the studies exclusively involved CHWs in COPD prevention and management for a healthy population, the population at risk, and COPD patients in Nepal. In the resource-constrained setting of Nepal, with a very low number of specialized health professionals in lung health, the involvement of CHWs remains a possible opportunity to curb the rising burden of COPD [26]. More than 50,000 female community health volunteers (FCHVs) are the CHWs in Nepal involved in community health programs for the last three decades. They serve as a link between government health services and local communities by imparting knowledge and awareness of health service availability, disease prevention, and health promotion [27, 28]. However, there is no evidence regarding the mobilization of FCHVs in the prevention and management of COPD in Nepal. There are important prospects of training and mobilizing FCHVs in curtailing the burgeoning burden of COPD in Nepal.

This article outlines the study protocol on the mobilization of FCHVs in public health activities to raise awareness for COPD prevention and its progression, called the Community-based intervention for prevention and management of COPD in Nepal (COBIN-P). This is a cluster randomized controlled trial (cRCT) aimed to evaluate the effect of a community-based home health educational intervention delivered by FCHVs on the prevention and management of COPD.

## Methods

This study protocol has been reported in accordance with the Standard Protocol Items: Recommendations for Clinical Interventional Trials (SPIRIT) guidelines (Additional file 1).

### Trial Design

A community-based, two-armed, open-label cluster-randomized controlled trial will be conducted in the semi-urban area of the Kaski district of western Nepal. It will be a parallel-group superiority trial with an allocation ratio of 1:1. The intervention time will be 12 months with assessments of outcomes at baseline and follow-up of the study.

### Study Settings

This study will be conducted in the semi-urban area of Pokhara (formerly known as Lekhnath Municipality). The study site is located in the Kaski district of western Nepal and is home to 58,816 residents, according to the 2011 census [10]. Similarly, 85% of the total population are literate, and the average life expectancy is 59.7 years [10]. It was administratively apportioned into 15 units called wards. The district public health office of Kaski district has reported 123 FCHVs working inside the municipality. Out of the ten health facilities, the Sisuwa Hospital is the only one with a provision of medical doctors, while the rest are run by a senior auxiliary health worker or health assistant. These facilities offer diagnosis and treatment for communicable diseases, including acute respiratory infection, diarrhea, tuberculosis, maternal and child health services comprising antenatal care, immunization, along with family planning and reproductive health services and non-communicable diseases services under Packages of Essential Non-communicable disease [29]. Our study will be an extension of a pre-existing Community-based Management of Non-communicable Diseases in Nepal (COBIN) project [30]. COBIN was initiated in 2013 and had thus far successfully concluded two studies on community-based management of hypertension and diabetes [31, 32]. No community-based interventions for COPD at the population level have been carried out in this study area to date.

### Study population

**Fig 1:**
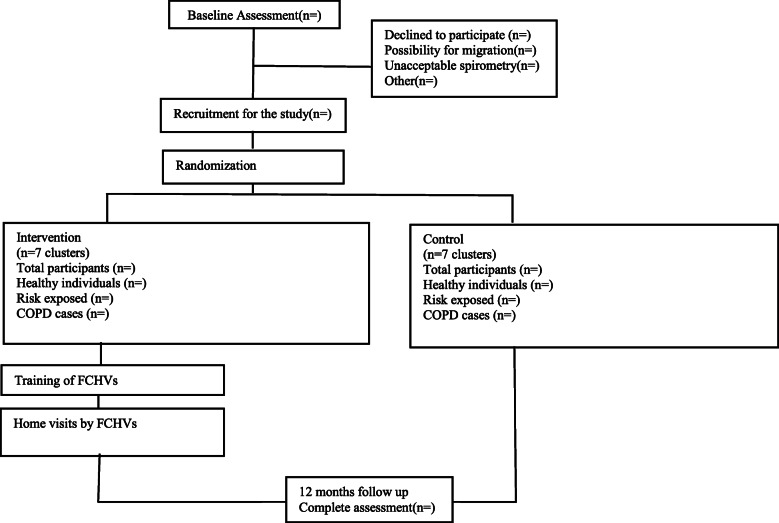
Planned flow of participants through the trial. *FCHV* Female Community Health Volunteer

The study population is individuals aged 40 years and above in the municipality is 15,507 as per the latest census data. Firstly, the baseline information will be collected in all 15 wards. During the baseline survey, people living with COPD and participants at risk of COPD and a healthy population will be identified and recruited for the trial (Fig. 1).

### Clusters

The 15 wards of the study area will constitute our clusters, but only 14 will be selected for the trial. We will randomly exclude one cluster by lottery method by an independent statistician. Allocation of the clusters into intervention and control arms is detailed in the section on randomization.

### Inclusion and exclusion criteria

Individuals aged 40 years and above who are listed in the voter list of 2007 as full-time residents of the study area are eligible for inclusion in the baseline survey. Those who can perform spirometry and provide the acceptable and reproducible spirometry graph to evaluate COPD will be included in the study intervention. Individuals, who are severely ill, who are unlikely to remain in the community throughout the intervention, who are pregnant women, who decline consent and have contraindications for conducting spirometry will be excluded from the study. The contradictions for spirometry include having active pulmonary tuberculosis or being on medications for pulmonary tuberculosis, thoracic, abdominal, or eye surgery in the last 6 months, history of mental illness, myocardial infarction in past 8 weeks prior to the study, and hospital admission in the past 6 months for cardiac illness [33].

### Sample size calculation

In the absence of a relevant Nepali study to take reference from for prevalence study, the sample size for this study is estimated according to the method suggested in the WHO STEPwise approach assuming the prevalence of COPD in Nepal to be 8% (a figure obtained from a Chinese study [34] and global burden of disease study based projection for Nepal [35]). When stratified by age group and sex (altogether eight groups in both sexes: 40–49, 50–59, 60–69, and ≥ 70), the sample size is 1508, when considering a 5% margin of error and expected response rate of 90%. Likewise, taking reference from a Chinese trial on community-based management of COPD for the expected mean difference between intervention and control arm as 19 ml FEV_1_ with a design effect of 1.83, considering an intracluster correlation of 0.01, the number of clusters per arm being 7, total sample size per arm will be 476 [36]. The coefficient of variation 0.45 was taken based on a previous study conducted in the same population [37]. Thus, the sample size required in the intervention for 80% of power and allowing 20% loss to follow-up will be 1143.

### Survey tools

The survey tool includes information on socio-demographic information, behavioral information, and relevant questions on respiratory conditions. Spirometry will accompany the questionnaire survey. Data enumerators with minimum qualification of a bachelor’s degree in health sciences will be trained to measure lung function using diagnostic portable spirometers and conducting an interview using the survey questionnaire.

The questionnaire to be used in the baseline and follow-up will be adapted from the WHO STEPwise Surveillance tool and similar past studies that measured the burden of lung disease and its risk factors [34, 36, 38–42]. St. George’s respiratory questionnaire, which has been adapted and validated for use in Nepal, will also be used for COPD patients [43]. The socio-demographic information includes age, sex, education, occupation, and income. Lifestyle variables comprise detailed smoking history (current/former, type of tobacco use, intensity, and duration), environmental exposure to tobacco smoke at home or workplace, household fuel type use, and exposure to biomass fuel. Participants’ blood pressure, weight, and height will be measured, and body mass index calculated.

### Detection of airflow obstruction

The lung function measurement will be made using the diagnostic portable spirometer (ndd Medical Technologies EasyOne Air^TM^ Medizintechnik, Zurich, Switzerland) preferred for its accuracy, handiness, and calibration stability. Forced vital capacity (FVC) and forced expiratory volume in 1 second (FEV_1_) will be used as parameters to assess the degree of airflow obstruction and to make the diagnosis and severity of COPD. The airflow obstruction will be measured in terms of FEV_1_/FVC compared against the lower limit of normal (LLN) and GOLD criteria. This measure means that the ratio of FEV_1_/FVC below LLN or FEV_1_/FVC below 0.70 of predicted normal will be considered indicative of airflow obstruction. Population-specific reference equations are not yet available for Nepal, so reference values from the North Indian population will be taken [44].

American Thoracic Society (ATS) and European Respiratory Society (ERS) task force guidelines for spirometry will be adhered to, and the interviewers will be trained in following the guidelines [33]. Participants will be seated down upright in a chair and then asked to draw in as much air as possible and then hinted to release all the air as quickly as possible inside the spirometer mouthpiece for at least 6 s. A spirometry measurement will be considered to meet ERS/ATS criteria for acceptability and reproducibility, including having at least three attempts, two of which must be acceptable with a difference between the largest and second-largest values of < 150 ml for both FEV_1_ and FVC. The spirometer will be calibrated daily with a 3 L syringe, and each spirometry report will be printed and reviewed by a research investigator or physician, and if found unacceptable, the procedure will be repeated.

Those individuals demonstrating obstruction during the pre-bronchodilator test will be given a short-acting bronchodilator through an inhaler, followed by spirometry after 10 to 15 min. Those participants who demonstrate FEV_1_/FVC<LLN or FEV_1_/FVC< 0.70 of predicted normal during postbronchodilator spirometry will then be identified as COPD cases.

### Randomization

The randomization approach, followed by the COBIN first trial on hypertension in Lekhnath Municipality, will be repeated in this study [30]. Out of 15 wards (clusters) in the municipality, one will be randomly excluded before randomization by lottery method. The remaining 14 clusters will be randomly assigned to the intervention and control group in 1:1 allocation ratio. The estimated sample size for the intervention study will be 1143. To include the study participants from every different sized clusters (wards), the required sample size of 1143 will be multiplied by the proportion of eligible participants in the ward. Then the intervention participants from each ward will be randomly selected. Research assistants will be blinded to the participants’ allocation status. To avoid selection bias following measures will be taken: (a) the randomization of the clusters will be done after the baseline survey, (b) clusters will not be withdrawn or added to the study, and (c) an independent epidemiologist will randomize the clusters.

### Recruitment procedures

The sampling frame of the COBIN study [30], including a population framework of all eligible participants using the election voter’s list for 2007, will be adapted. The COBIN study baseline survey used this voter list and prepared a list of eligible respondents to participate in the trial. From the framework, we will list all individuals aged 40 years and above. The voter list provides information on name, age, household number, and address. Simple random sampling will be applied to select households. Kish method will be applied if more than one eligible participant from the same household exists during data collection [45]. During the baseline survey, participants will be identified and requested to participate in the study by the data enumerators at the participant’s household. After completing the survey, eligible participants will be randomly selected proportionately, with cluster size meeting the required sample size of 1143. On the final day of the training, FCHVs from each cluster will sit together and allocate the number of households to be visited during the intervention.

### Study intervention

Following randomization of the clusters, FCHVs falling in the intervention arm will receive six days of training on COPD, prevention, and management. Based on information from the baseline survey FEV_1_ and FEV_1_/FVC measures, participants will be categorized into two categories: healthy individuals, those at risk of COPD, and COPD patients. The interventions will then be designed accordingly, targeting each of these three groups. It includes home-based health education, tailored intervention, and referral for treatment, respectively, which FCHVs will deliver in three routine visits throughout the intervention period of 12 months. All three groups in the intervention arm will be exposed to home-based health education. Those at risk and those identified with COPD will additionally also be exposed to the tailored intervention, and those with COPD will also be actively motivated for treatment.

The home-based health education will include efforts from FCHVs: (1) to improve knowledge on COPD by providing basic information on COPD, its risk factors, symptoms, and its progression; (2) counseling the households on reducing and avoiding exposure to the risk factors (e.g., household air pollution and tobacco) of COPD; (3) ways of improving indoor air quality. Educational materials will be developed for their guidance with a focus on pictorial information. These materials will be developed in consultation with a pulmonologist, health education experts in the National Health Education and Information Centre of Government of Nepal, nurses, FCHVs, local health workers, patients living with COPD, and other stakeholders. The educational materials will be pretested among the FCHVs of Pokhara , a comparable area nearby not included in the study area.

The tailored intervention, which will target those at risk and those with COPD, will include the following: (1) information to avoid exacerbations and maintain general lung health (exposure reduction, exercise, sleep, and nutrition), (2) information on the hazards of cigarette smoking, (3) advice on smoking cessation, (4) suggestions for maintaining improved cooking stoves, and (5) better indoor cooking environment with adequate ventilation. Those who show willingness towards quitting smoking will be continually motivated by the FCHVs and followed up during their routine visits. For those with COPD, FCHVs will assess the COPD status using COPD status assessment guides for FCHVs (CAF guide) designed with basic questions on cough, breathing problems, and daily functioning activities. Thus, they recommend referring to hospitals, suggesting being physically active, and counseling on proper nutrition. In addition, FCHVs will also teach persons with COPD about the basic breathing techniques (purse lip breathing, deep breathing, and diaphragmatic breathing) added with stamina and endurance-building exercises.

### Usual care in control clusters

The usual care will include whatever prevention or treatment services are available at present. After 12 months of intervention, a follow-up survey will be conducted by trained data enumerators.

### Analysis

The primary and secondary aims of this study will be assessed using an intent-to-treat analysis. The primary outcome of the study will be the average difference in the rate of decline of FEV_1_ and FEV_1_/FVC between the intervention and control arms. We will use a mixed model ANOVA to test the statistical significance of the difference in the rate of change of primary outcomes between the intervention and control arms. Potential confounder variables statistically significantly associated with treatment assignment will be included in the multivariable analysis.

Our secondary outcomes include the change in the quality of life among people living with COPD, awareness, and knowledge on COPD, and change in magnitude of risk factors of COPD. The quality of life will be measured using the validated Nepali version of the St. George Respiratory Questionnaire [43]. The change in the quality of life will be assessed using the difference in the mean or median score at the beginning and end of the intervention accompanied by the standard deviation or interquartile range of the difference. The change in awareness and knowledge related to COPD will be assessed using the change in the proportion of people aware and knowledgeable regarding COPD. Similarly, the change in risk behaviors, including smoking and indoor cooking, will be assessed by measuring the proportion of individuals who changed those behaviors in the intervention and control clusters. The subgroup analysis will be performed based on COPD status, grade, and smoking status. Complete case analysis will be done for missing data less than 10%. In case of missing data for more than 10%, it will be addressed by the multiple imputation method. We will explain the effect of non-adherence and missing data on our results. Participants receiving three routine home visits by FCHVs during the intervention period will be considered complete adherence. We will assess two-way interaction among sex and treatment allocation, and also among smoking status and treatment allocation. STATA software (StataCorp, College Station, TX, USA) will be used for the data analysis of the study.

### Study outcomes

The primary outcome will be the rate of decline in FEV_1_ and FEV_1_/FVC ratio in the intervention and control groups from baseline and follow-up. The secondary outcomes will include the change in awareness and knowledge related to COPD, quality of life of COPD patients, modification of risk factors of COPD such as active and passive smoking and change in indoor cooking behavior from baseline between the intervention and control clusters. The questionnaire-based interview and spirometry during the baseline and follow-up survey will be conducted in participants’ households using a portable diagnostic spirometer by data enumerators trained for 3 weeks.

### Trial management

To ensure data quality, a robust mechanism will be set up. Firstly, the data enumerators will receive intensive training on data collection using spirometer and questionnaire. The principal investigator will monitor data enumerators on a day-to-day basis. The data entry assistants will inspect the data manually at first and enter the data in an EpiData software file. Ten percent of the data will be double entered to qualify potential errors in data entry. There will be cross-checking the data quality on the spot and in our field office for any incomplete, inconsistent, and invalid data. Two independent pulmonologists will further review the spirometry graph for quality control. Any deviation in the data quality will require retesting in the field.

Data safety and monitoring board (DSMB) is formed secretariat at Nepal Health Research Council consisting of five members completely independent of the investigators and have no financial, scientific, or other conflicts of interest with the trial. The chairman of the board convenes DSMB meetings as conference calls as well as in person. An emergency meeting of the DSMB may be called at any time if questions of study participants’ safety arise. The change or amendments in protocol will be reported to the ethical review board of Nepal Health Research Council, DSMB, FCHVs, trial registries, and journals with relevant publications. The trial sponsor is the Department of Public Health, Aarhus University (Bartholins Alle 2, 8000 Aarhus C, Denmark; +45-87167936), responsible for the overall management of research planning and implementation.

### Indemnities

The trial brings very negligible risk to the study participants, so we have no compensation or insurance plan for the participants. However, 5 US dollars per home visit will be given to FCHVs for covering their transportation expenses.

### Data integrity

Data integrity will be enhanced by having data collected by trained data enumerators with a health science background and adherence to assessment protocols.

### Minimization of contamination

To minimize the contamination during the intervention, both the risk and level of contamination will regularly be monitored among both FCHVs and study participants. FCHVs will be asked not to share the study information and not to provide any support to people beyond their assigned cluster during the pre-intervention intensive training and throughout the intervention period of 1 year. Likewise, the design of the cluster will be such that intervention and control clusters will be geographically divided; hence the chance for a regular meeting of participants from two different arms of the study will be very low. In addition, all the potential ways by which the information in the intervention group influences the control participants will be followed up. If found to influence the study significantly, those factors will be adjusted during the effect estimation.

### Intervention fidelity

The fidelity of the study will be guaranteed, starting from the pre-intervention training to FCHVs. They will be explained about the importance of enduring with all the constituents of intervention in the experimental arm. Similarly, FCHVs in the intervention arm will receive a register to record and report all activities. They will report to the project team every four months, and the field coordinator will meet regularly to monitor the FCHVs’ activities. Supervision checklists will be used to track and update the knowledge and skill levels of FCHVs. At the follow-up survey, the data enumerators will ask the participants about the visits and counseling made by FCHVs.

### Interim analyses and stopping rules

The study does not have an interim analysis plan, as we do not expect a situation that would lead us to study termination. However, the study will be temporarily stopped to assess the implications on the study design if an extreme situation occurs, such as a natural disaster, a conflict, or duplication of similar interventions in our control area.

### Dissemination of results

The study results will be shared through workshops, local and international conferences, seminars, and events organized with local stakeholders. We plan to publish the results of this trial as a peer-reviewed publication.

## Discussion

WHO data show that over 90% of deaths from COPD occur in low- and middle-income countries [2]. Most of this is attributable to air pollution (indoor and outdoor) and tobacco smoke. These are the very countries that face significant challenges in spreading awareness of chronic lung diseases and their risk factors among the public and decision-makers. Additionally, access to clinically efficient and cost-effective interventions and the provision of diagnosis and treatment services are inadequate. Therefore, there is an urgent need to develop suitable solutions to the local context and, at the same time, feasible in terms of cost and clinical effectiveness. In a resource-constrained setting like Nepal, with very limited health service provision on chronic disease prevention and management, especially on chronic respiratory diseases like COPD. The approach for task-sharing to community health workers could be one of the best options. Mobilizing community health workers, i.e., FCHVs in Nepal, have shown promising results in other public health issues and chronic disease prevention and management like hypertension and diabetes [31, 32]. This study capitalizes on the experience, knowledge, and skills of FCHVs in the prevention and management of COPD. This approach is expected to serve as a potential way to address the neglected issue of COPD in Nepal. Its success will also potentially serve as a guide to national scale-up and similar efforts in low- and middle-income countries elsewhere.

## Trial status

**Fig. 2:**
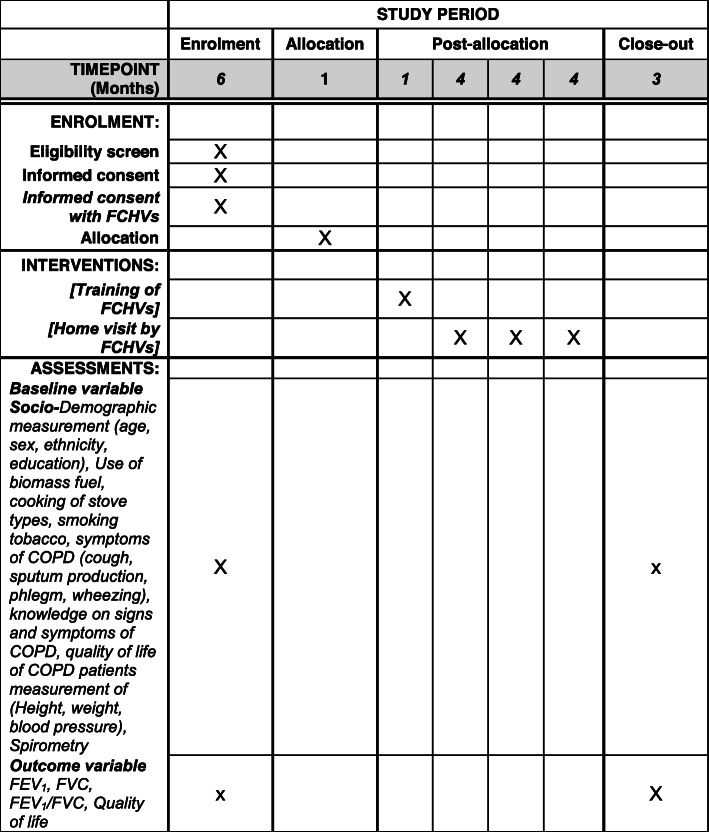
Schedule of enrolment, interventions, and assessments (SPIRIT figure)

The trial has been closed to participant recruitment, but the trial is ongoing. The trial recruitment began on 1 May 2019 and was completed on 28 September 2019. At the end of March 2021, we will complete the endpoint assessment of all the participants. At the end of April 2021, we will complete the outcome assessment of all the participants. The schedule of enrollment, interventions, and assessments is presented in Fig. 2. The trial protocol has been submitted for publication after the end of recruitment due to a delay in finalizing the manuscript affected by the author’s travel plan for fieldwork considering the tight timeline of the Ph.D. study of the principal investigator. However, the study protocol was registered to clinicaltrials.gov before starting the baseline survey.

The protocol version number and date: V1, 16 April 2020.

## Data Availability

Final trial data are available upon reasonable request to the principal investigator of the study.

## Abbreviations

ATS: American Thoracic Society
CHWs: Community Health Workers
COPD: Chronic obstructive pulmonary diseases
COBIN: Community-based Management of Non-communicable Diseases in Nepal
cRCT: Cluster randomized controlled trial
DSMB: Data safety and monitoring board
ERS: European Respiratory Society
FCHVs: Female Community Health Volunteers
FEV_1_: Forced expiratory volume in 1 s
FVC: Forced vital capacity
GOLD: Global Initiative for Chronic Obstructive Lung Disease
LMICs: Low- and middle-income countries
NCDs: Non-communicable diseases
SPIRIT: Standard Protocol Items: Recommendations for Interventional Trials
WHO: World Health Organization
